# Online platform for applying space–time scan statistics for prospectively detecting emerging hot spots of dengue fever

**DOI:** 10.1186/s12942-016-0072-6

**Published:** 2016-11-25

**Authors:** Chien-Chou Chen, Yung-Chu Teng, Bo-Cheng Lin, I-Chun Fan, Ta-Chien Chan

**Affiliations:** 1 Center for Geographic Information Science, Research Center for Humanities and Social Sciences, Academia Sinica, 128 Academia Road, Section 2, Nankang, Taipei, 115 Taiwan, ROC; 2 Institute of History and Philology, Academia Sinica, 128 Academia Road, Section 2, Nankang, Taipei, 115 Taiwan, ROC

**Keywords:** Scan statistics, Dengue fever, Real-time, Spatio-temporal, Flexibility

## Abstract

**Background:**

Cases of dengue fever have increased in areas of Southeast Asia in recent years. Taiwan hit a record-high 42,856 cases in 2015, with the majority in southern Tainan and Kaohsiung Cities. Leveraging spatial statistics and geo-visualization techniques, we aim to design an online analytical tool for local public health workers to prospectively identify ongoing hot spots of dengue fever weekly at the village level.

**Methods:**

A total of 57,516 confirmed cases of dengue fever in 2014 and 2015 were obtained from the Taiwan Centers for Disease Control (TCDC). Incorporating demographic information as covariates with cumulative cases (365 days) in a discrete Poisson model, we iteratively applied space–time scan statistics by SaTScan software to detect the currently active cluster of dengue fever (reported as relative risk) in each village of Tainan and Kaohsiung every week. A village with a relative risk >1 and *p* value <0.05 was identified as a dengue-epidemic area. Assuming an ongoing transmission might continuously spread for two consecutive weeks, we estimated the sensitivity and specificity for detecting outbreaks by comparing the scan-based classification (dengue-epidemic vs. dengue-free village) with the true cumulative case numbers from the TCDC’s surveillance statistics.

**Results:**

Among the 1648 villages in Tainan and Kaohsiung, the overall sensitivity for detecting outbreaks increases as case numbers grow in a total of 92 weekly simulations. The specificity for detecting outbreaks behaves inversely, compared to the sensitivity. On average, the mean sensitivity and specificity of 2-week hot spot detection were 0.615 and 0.891 respectively (*p* value <0.001) for the covariate adjustment model, as the maximum spatial and temporal windows were specified as 50% of the total population at risk and 28 days. Dengue-epidemic villages were visualized and explored in an interactive map.

**Conclusions:**

We designed an online analytical tool for front-line public health workers to prospectively detect ongoing dengue fever transmission on a weekly basis at the village level by using the routine surveillance data.

## Background

Cases of dengue fever (DF) have increased in areas of Southeast Asia in recent years. For example, Singapore has experienced increasing DF incidence over the past 40 years [1]. Similarly, Taiwan suffered from its largest outbreak in 2014 [2], and the number of cases has hit a record-high 42,856 in 2015, with the majority in Tainan and Kaohsiung, two metropolitan areas in southern Taiwan.

According to Taiwan Centers for Disease Control (TCDC), insecticide spraying operations are carried out as the epidemic emergency-handling mechanism of DF [3]. To guide the implementation of insecticide spraying, an effective monitoring system is required to promptly report the progress of an epidemic and initiate a spraying plan in the targeted neighborhood [4].

Scan statistics have proven to be useful in detecting spatiotemporal clusters of diseases [5–7]. Although prospective scan statistics have been applied in detecting ongoing transmission of malaria [8], shigellosis [9, 10], and Campylobacter [5], what health workers might be interested in is the ability to relate existing hot spots to future infection [8, 11]. Taking DF as an example, since DF is an acute transmission disease, health workers might not only want to identify the hot spots in week *t*, but also be interested in the duration of the identified hot spots, which is important for them to plan the insecticide spraying campaign for the coming 1 (${\text{week}}_{t + 1}$) or 2 (${\text{week}}_{t + 2}$) weeks. On the other hand, calibrating proper parameters such as spatial and temporal windows in scan statistics is difficult for non-expert users, and requires a process of model tuning. And few studies have rationalized the parameter specifications [7, 12]. Moreover, with the advance in online techniques and utilization of mobile phones, an online scan statistics tool might help public health workers detect ongoing transmission promptly, compared to the current desktop software.

To implement effective intervention, embedding an outbreak detection tool in a user-friendly platform is essential. Leveraging scan statistics and geo-visualization techniques, we aimed to design an online analytical tool for local public health workers to identify ongoing DF transmission at the village level using routine surveillance data. Sensitivity and specificity analysis comparing the scan-based detections with the true results from the historical surveillance data were conducted in two cities of Taiwan. We look to gain insights that can guide and prioritize future insecticide spraying of DF prevention practices in the community.

## Methods

### Study area

**Fig. 1:**
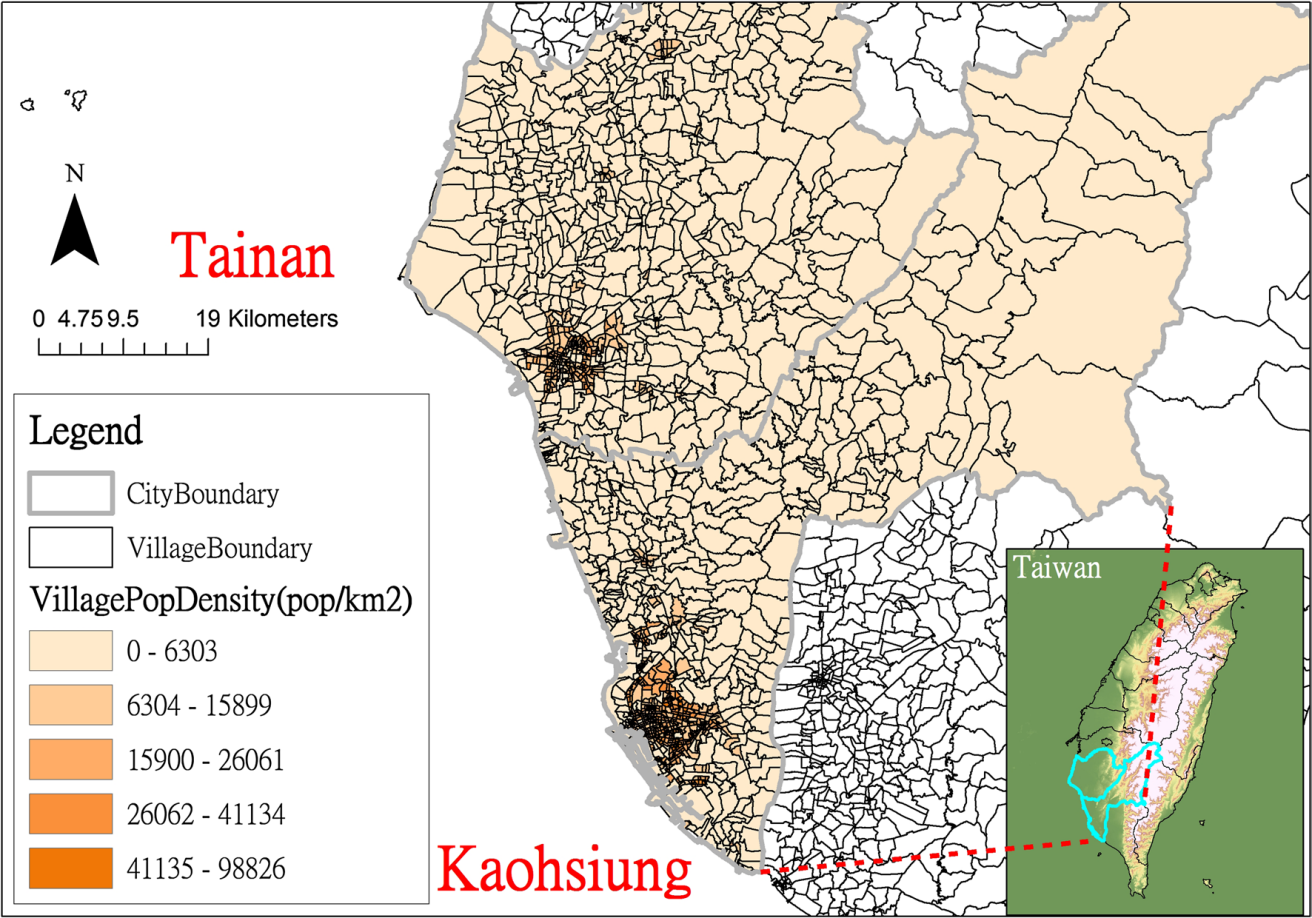
The study areas: Tainan and Kaohsiung, Taiwan

The Tropic of Cancer cuts across Taiwan and divides this island into tropical and subtropical zones. Tainan and Kaohsiung, located south of the Tropic of Cancer, span 2191 and 2952 km^2^ respectively and include both urban and rural communities (Fig. 1). In 2015, the total population was 1.88 million in Tainan and 2.78 million in Kaohsiung.

### Data

A confirmed DF case is defined as a person who resided in Tainan or Kaohsiung during January 1, 2014–December 31, 2015 and was infected by DF in Taiwan. The datasets of a total of 57,516 confirmed indigenous DF cases in the study areas were obtained from the Taiwan Open Data Platform (http://data.gov.tw/). Individual information, such as age, date of onset, and x- and y-coordinates (centroid) of the affiliated basic statistical areas (BSAs) [13], was also included. Other demographic information, including age stratified population counts at the village level, was obtained from the Ministry of Interior, Taiwan (http://210.65.89.57/STAT/Web/Platform/STAT_PlatformHome.aspx).

### Scan statistics

The free SaTScan software applies scan statistics [14] to compare the disease risk within and outside the scanning window. The discrete Poisson model with no covariate, which is the reference model in the study, assumes the expected number of cumulative DF cases in each village is proportional to its population size. To incorporate demographic information in a discrete Poisson model, we replaced the raw population count with the expected number of 1-year cumulative cases ($t_{0} \sim{\text{week}}_{t}$) at each village *i* in scan statistics. The expected number of cumulative case for the covariate adjustment model was estimated from the following Poisson regression:

$$\log \left( {{\text{cumulative}}\; {\text{case}}_{it} } \right) = \alpha + \beta_{1}\, {\text{population}}\;{\text{at}}\;{\text{risk}}_{i} + \beta_{2}\, {\text{working}}\;{\text{age}}\;{\text{proportion}}_{i}$$

where *i* indicates a village *i* in the study areas and *t* denotes week *t* during 2014–2015.

We assumed that the total population at risk at village *i* and the proportion of working-age people (ages of 15–64) at village *i* were associated with the expected cumulative cases at each village *i* [15]. A space–time cluster related to the proportion of working age in a village might be occurred due to the journey to work patterns.

**Fig. 2:**
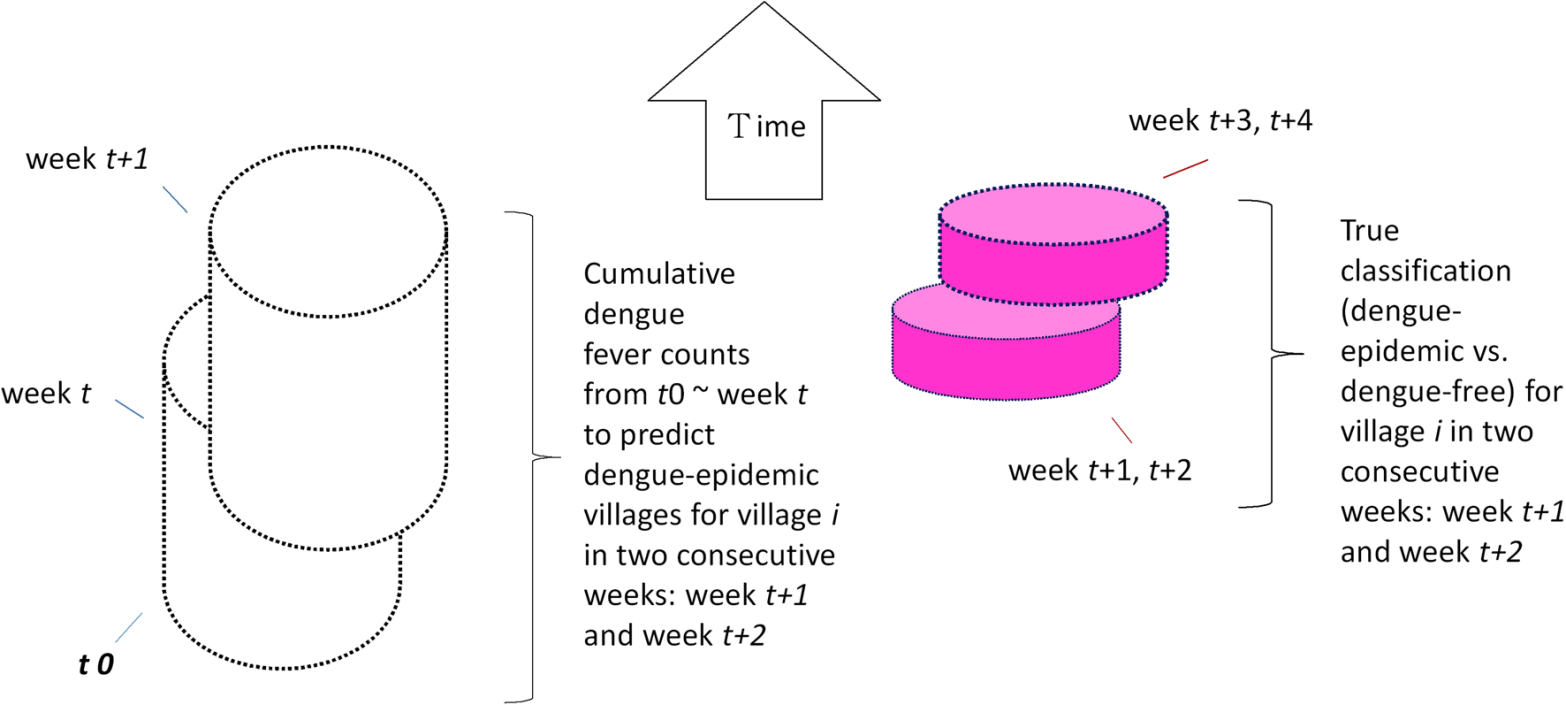
Design of the study: the moving window approach to prospectively detect spatiotemporal hotspots

The prospective space–time statistic, defined by a cylindrical window (Fig. 2) with a circular geographic base and with height corresponding to time ($t_{0} \sim{\text{week}}_{t}$: 365 days in the study) [16], was executed weekly (the moving window approach) as if it had been performed in 2014–2015 to assess the relative risk of DF in week *t*. The time interval in this study is 1 week (7 days), and SaTScan uses Monte Carlo hypothesis testing to generate *p* values and recurrence intervals (1/*p* weeks) for villages with excess risk. We reported the first most likely cluster and secondary clusters if *p* value <0.05 with no geographical overlap.

Instead of arbitrary specification, we used a trial-and-error approach to explore SaTScan’s parameters, including the maximum spatial window (25 and 50% of the population at risk), the maximum temporal window (14 and 28 days), and *p* values (<0.05 and <0.001). The initial value of 14 days for the temporal widow was based on the 2-week extrinsic incubation period of DF [17, 18]. The effects of different combinations of parameters on cluster detection can be observed through the sensitivity analysis. To routinely launch the program, we executed SaTScan from rsatscan, an R package (http://www.R-project.org/) which runs SaTScan in the operating system.

### Sensitivity and specificity

**Fig. 3:**
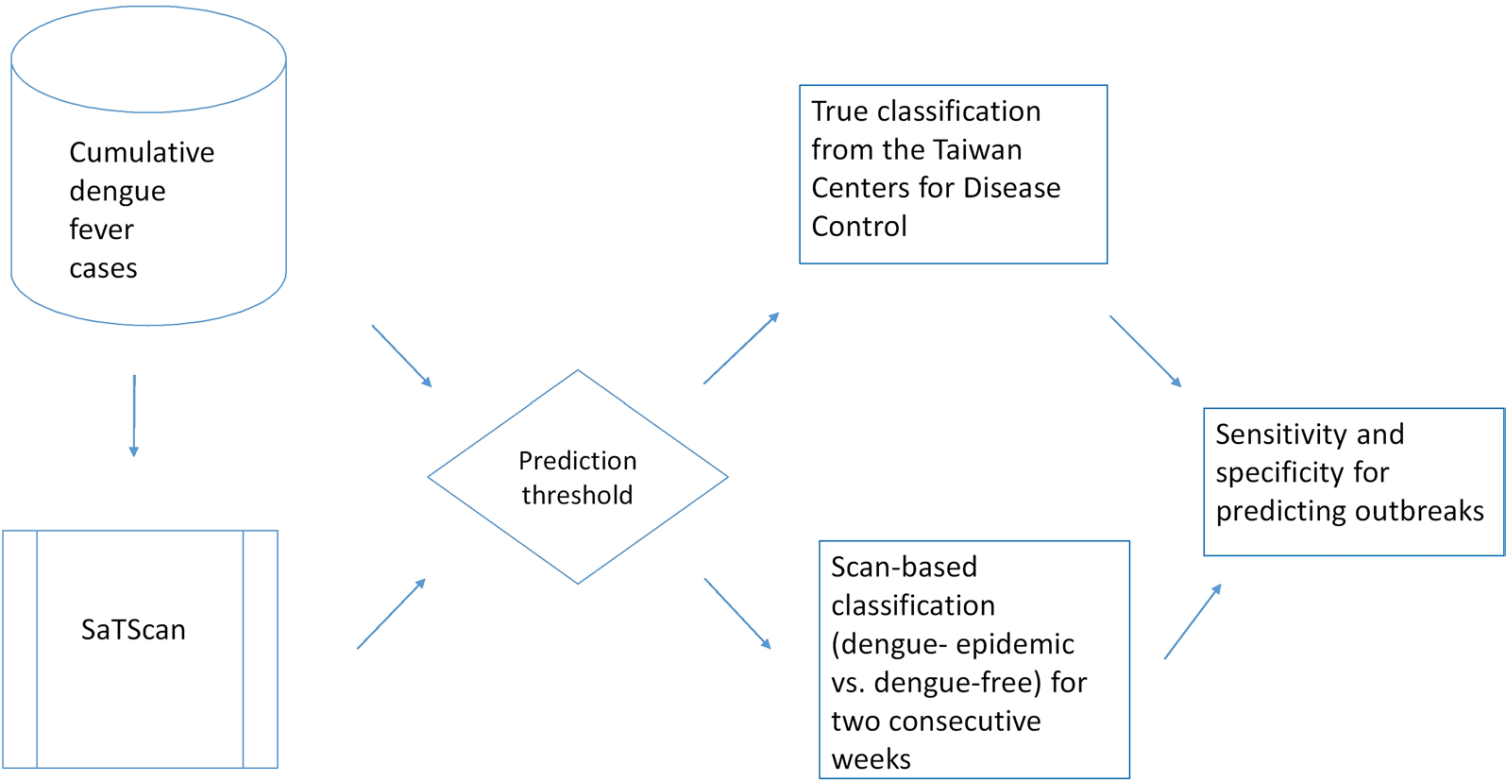
Flowchart of the study

We are interested in whether or not the cumulative case information of a village from $t_{0}$ to ${\text{week}}_{t}$ could be related to the risk of DF for two consecutive weeks ${\text{week}}_{t + 1 } ,\;{\text{week}}_{t + 2}$ (i.e., a 2-week threshold) based on scan statistics (Fig. 3). To evaluate our model, the following steps were operated weekly (with a total of 92 iterations) in the study.

- *Step 1* a village *i* with a relative risk >1 and *p* value <0.05 (estimated from SaTScan) using DF cumulative counts from $t_{0}$ to ${\text{week}}_{t}$ was identified as a dengue-epidemic village (vs. a dengue-free village).
- *Step 2* we dichotomically classified the village *i* as dengue-epidemic (vs. dengue-free) if its cumulative case numbers during ${\text{week}}_{t + 1 }$ and ${\text{week}}_{t + 2}$ were ≥1, according to TCDC’s historical DF surveillance statistics.
- *Step 3* we estimated the sensitivity and specificity for detecting 2-week outbreaks in the study areas weekly by comparing the scan-based classification from the step (1) with the true classification from the step (2).

The sensitivity for detecting 2-week outbreaks in week *t* in the study is defined as:

$${\text{Sensitivity}}_{t} = \frac{{\# \;{\text{of}}\;{\text{dengue-epidemic }}\;{\text{villages}}\;{\text{identifed}}\;{\text{by}}\;{\text{SaTScan}}\;{\text{in}}\;{\text{week}}_{t} }}{{\# \;{\text{of}}\;{\text{villages}}\;{\text{in }}\;{\text{week}}_{t + 1,t + 2} \;{\text{where}} \;{\text{case}} \ge 1}}$$

The specificity for detecting 2-week outbreaks in week *t* in the study is defined as:

$${\text{Specificity}}_{t} = \frac{{\# \;{\text{of }}\;{\text{dengue-free}}\; {\text{villages}}\; {\text{identifed}}\; {\text{by}}\; {\text{SaTScan}}\; {\text{in }}\;{\text{week}}_{t} }}{{\# \; {\text{of }}\;{\text{villages}}\; {\text{in}}\; {\text{week}}_{t + 1,t + 2} \; {\text{where}}\;{\text{case}} = 0}}$$

Both sensitivity and specificity range from 0 to 1. A higher sensitivity indicates that scan statistics precisely detect the 2-week outbreak of DF in village *i*. On the other hand, a greater specificity shows that scan statistics accurately indicate the absence of DF outbreak in village *i* for the next 2 weeks. A sensitivity analysis examining a strict detection duration threshold (ongoing transmission only extending to the next week) was also conducted.

### Web GIS (Geographical information system)

**Fig. 4:**
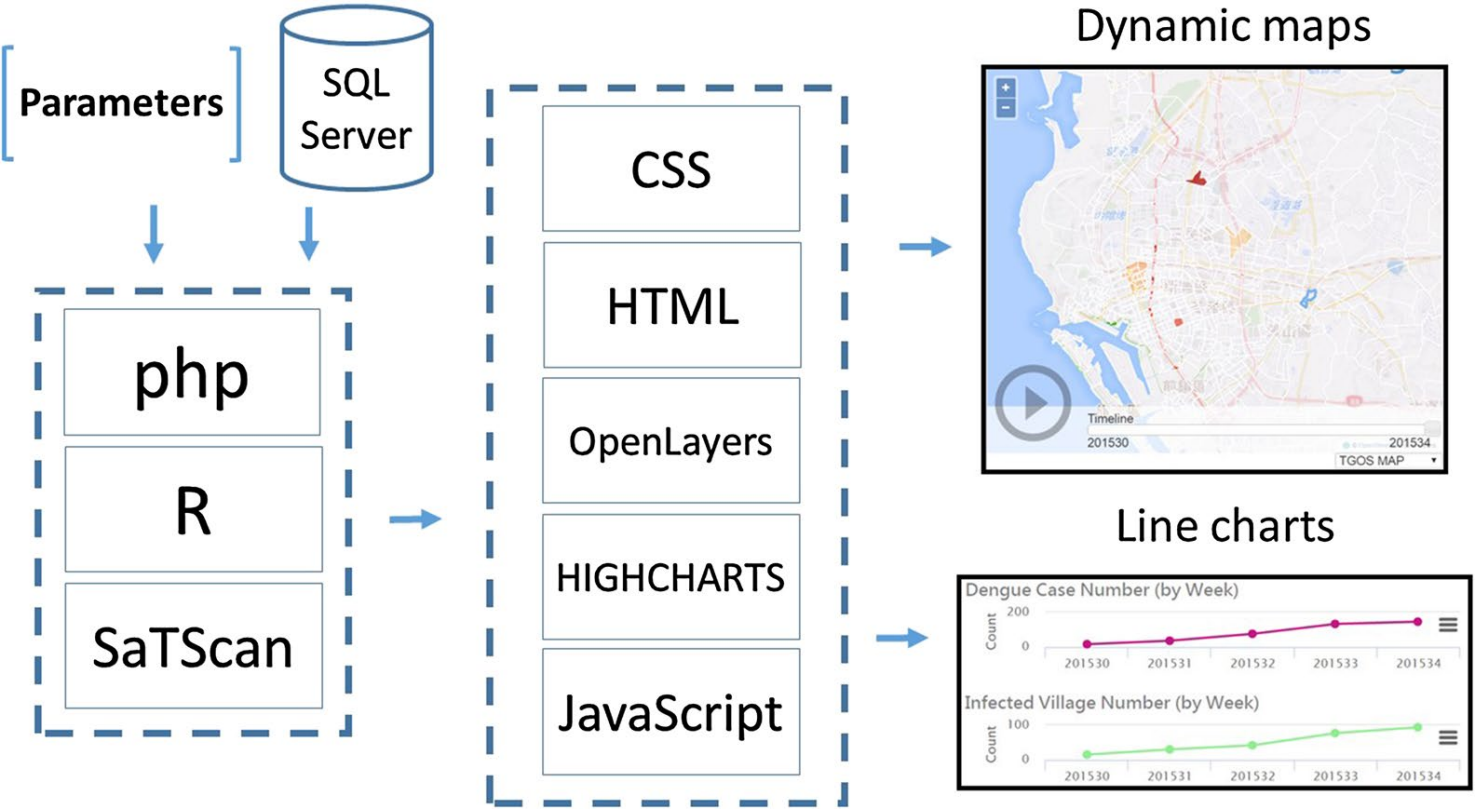
Online platform design

**Fig. 5:**
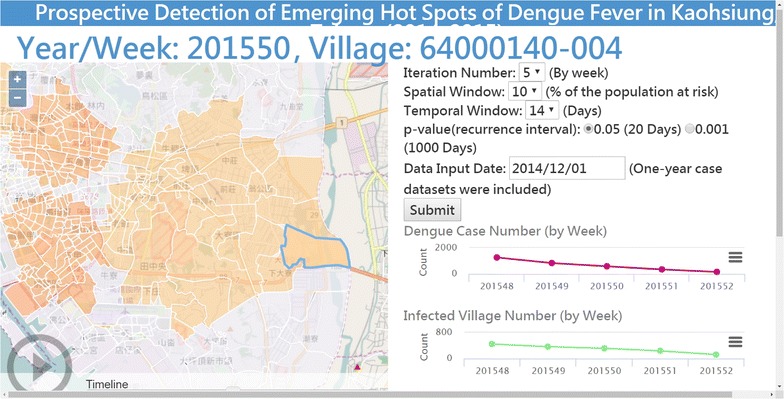
Online platform snapshot (http://scan.geohealth.tw)

An online analytical platform, powered by PHP (version 5.5), JavaScript (OpenLayers and Highcharts libraries), and HTML, provided an interactive interface for users to manipulate relevant parameters of scan statistics and visualize the weekly relative risk of DF at the village level (Fig. 4). First, SaTScan-related parameters, like the maximum spatial size in the total population at risk, were passed to R through PHP for each simulation. Second, we executed R’s rsatscan package in batch mode via PHP. Third, the SaTScan estimates, like the relative risk of DF in each village, were passed to JavaScript by PHP. Finally, line charts, tables, and maps were visualized via HTML and JavaScript libraries. The web service is available at http://scan.geohealth.tw (Fig. 5).

## Results

To prospectively detect the ongoing transmission of DF at the village level, we used the confirmed indigenous dengue cases (*n* = 57,516) in Tainan and Kaohsiung between January 1, 2014 and December 31, 2015 (Table 1). There are a total of 752 and 891 villages in Tainan and in Kaohsiung, respectively (Fig. 1). Overall, the average population density is around 9655 inhabitants/km^2^ in the study areas.

**Table 1:** Description of indigenous dengue fever cases during 1/1/2014–12/31/2015 in Tainan and Kaohsiung, Taiwan

	Case numbers in Tainan (%)	Case numbers in Kaohsiung (%)
Sex		
Male	11,367 (49.6)	17,187 (49.6)
Female	11,509 (50.4)	17,453 (50.4)
Age		
0–14	1786 (7.8)	2909 (8.4)
15–64	16,305 (71.2)	25,392 (73.3)
≥65	4785 (21.0)	6339 (18.3)
Total	22,876 (100.0)	34,640 (100.0)

**Fig. 6:**
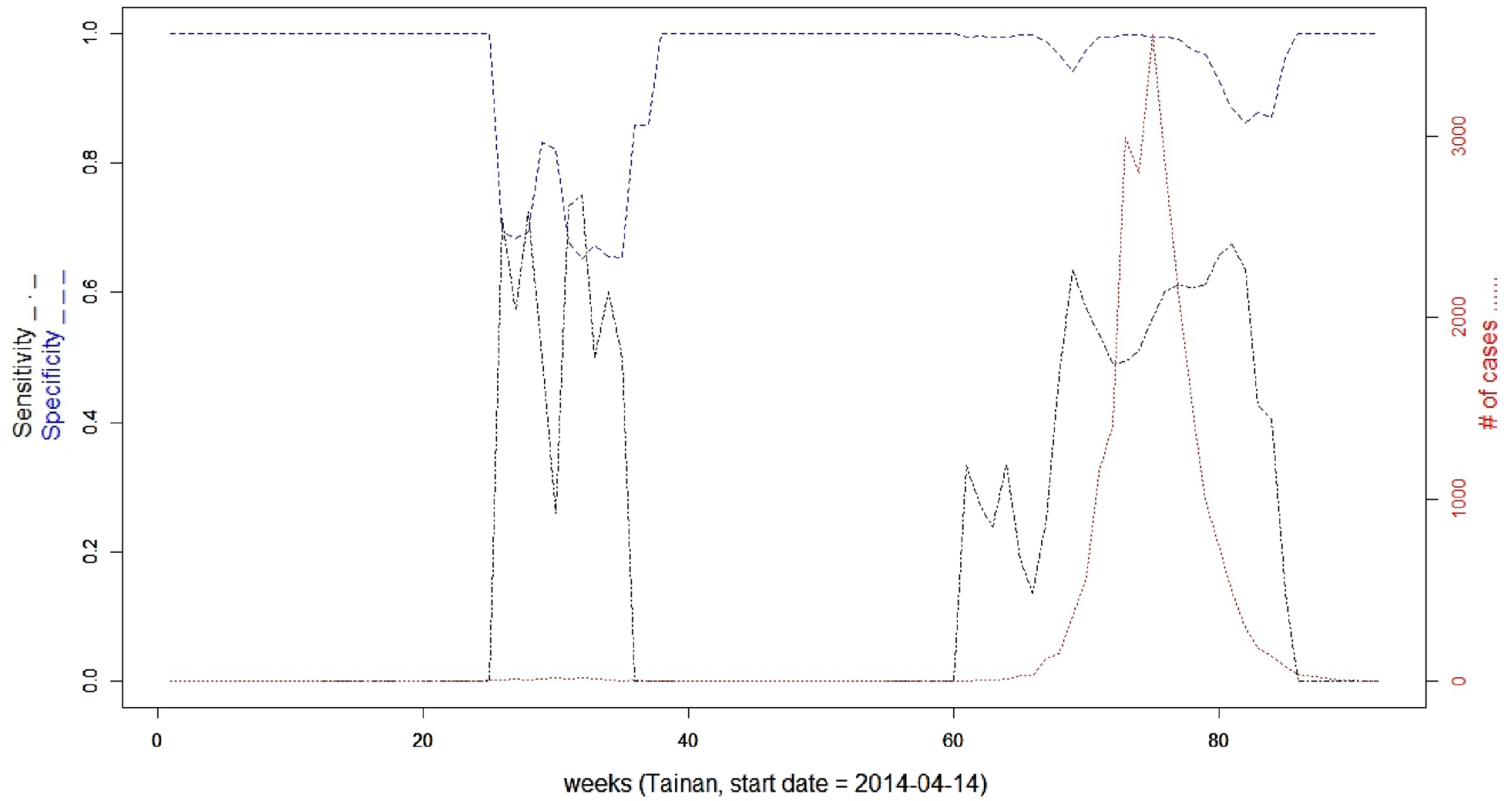
Sensitivity and specificity for outbreak detection in Tainan, Taiwan with the covariate adjustment model (maximum spatial window = 50% of the total population at risk; maximum temporal window = 28 days; *p* value <0.001; detection duration threshold = 2 weeks)

**Fig. 7:**
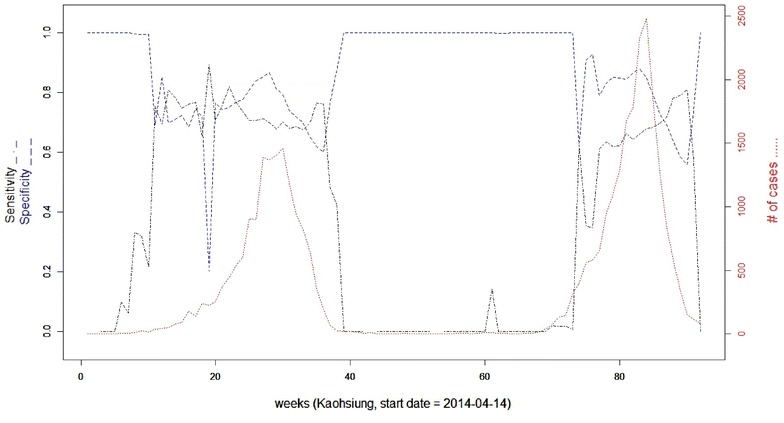
Sensitivity and specificity for outbreak detection in Kaohsiung, Taiwan with the covariate adjustment model (maximum spatial window = 50% of the total population at risk; maximum temporal window = 28 days; *p* value <0.001; detection duration threshold = 2 weeks)

As the epidemic curve illustrates (Fig. 6), the outbreak of DF started in the 70th week and peaked in the 75th week in Tainan. For Kaohsiung, we observed two peaks of DF epidemic, climaxing in the 30th week and 84th week (Fig. 7). To evaluate the detection capability, different model specifications (reference against covariate adjustment), percentages of the total population at risk (the maximum spatial window), and lengths of the maximum temporal window were manipulated in SaTScan (Tables 2, 3). Overall, the sensitivity for outbreak detection increases as the number of cases grows. However, an inverse relationship is identified between sensitivity and specificity during an epidemic (Figs. 6, 7). On average, the sensitivity and specificity for outbreak detection given a weekly number of cases ≥500 are around 0.569 and 0.982 respectively, as the maximum spatial and temporal windows were set to 50% of the total population at risk and 28 days respectively in Tainan for the covariate adjustment model with *p* value <0.001 (recurrence interval = 1000 weeks) (Fig. 6). Correspondingly, the mean sensitivity and specificity of detections are around 0.661 and 0.800 respectively for the counterpart of Kaohsiung (Fig. 7).

**Table 2:** Sensitivity analysis of parameters (spatial/temporal/*p* value) by location as the number of cases ≥500 per week

Model	Detection duration threshold (weeks)	Spatial (%)/temporal (days)/*p* value	Mean sensitivity (total elapsed time^a^ (s))	
			Tainan	Kaohsiung
Reference	2	(25, 14, 0.05)	0.680 (490.10)	0.631 (750.64)
		(50, 28, 0.05)	0.580 (1123.61)	0.652 (1305.84)
		(25, 14, 0.001)	0.662 (472.80)	0.626 (745.11)
		(50, 28, 0.001)	0.578 (1139.66)	0.652 (1196.82)
	1	(25, 14, 0.05)	0.705 (453.06)	0.656 (770.58)
		(50, 28, 0.05)	0.656 (1077.49)	0.690 (1221.14)
		(25, 14, 0.001)	0.689 (479.27)	0.651 (730.03)
		(50, 28, 0.001)	0.654 (1079.73)	0.690 (1262.36)
Covariate adjustment	2	(25, 14, 0.05)	0.636 (481.78)	0.629 (777.33)
		(50, 28, 0.05)	0.568 (1221.47)	0.661 (1251.64)
		(25, 14, 0.001)	0.618 (507.71)	0.629 (745.58)
		(50, 28, 0.001)	0.569 (1154.70)	0.661 (1223.97)
	1	(25, 14, 0.05)	0.678 (513.56)	0.655 (729.69)
		(50, 28, 0.05)	0.645 (1023.12)	0.701 (1365.97)
		(25, 14, 0.001)	0.661 (494.28)	0.654 (743.39)
		(50, 28, 0.001)	0.644 (1080.50)	0.701 (1296.22)

^a^Testing environment: Windows Server 2012; Intel Xeon E5-2630 v3 @ 2.4 GHz 4 cores; RAM = 32 GB

**Table 3:** Specificity analysis of parameters (spatial/temporal/*p* value) by location as the number of cases ≥500 per week

Model	Detection duration threshold (weeks)	Spatial (%)/temporal (days)/*p* value	Mean specificity	
			Tainan	Kaohsiung
Reference	2	(25, 14, 0.05)	0.597	0.714
		(50, 28, 0.05)	0.980	0.785
		(25, 14, 0.001)	0.619	0.718
		(50, 28, 0.001)	0.981	0.785
	1	(25, 14, 0.05)	0.573	0.657
		(50, 28, 0.05)	0.957	0.721
		(25, 14, 0.001)	0.596	0.662
		(50, 28, 0.001)	0.959	0.721
Covariate adjustment	2	(25, 14, 0.05)	0.736	0.651
		(50, 28, 0.05)	0.982	0.800
		(25, 14, 0.001)	0.755	0.652
		(50, 28, 0.001)	0.982	0.800
	1	(25, 14, 0.05)	0.713	0.610
		(50, 28, 0.05)	0.961	0.733
		(25, 14, 0.001)	0.732	0.611
		(50, 28, 0.001)	0.961	0.733

Regarding the maximum spatial and temporal windows, we found that the mean sensitivity for detecting outbreaks rises in Kaohsiung but falls in Tainan as the combination of the maximum spatial and temporal parameters varies from {25%, 14 days} to {50%, 28 days} (Table 2). The cost is the rise in computation time for the total 92 iterations (Table 2). On the other hand, as the values of parameter combinations increase (Table 3), the specificity for detecting outbreaks increases in both areas.

Regarding the sensitivity analysis on the detection duration threshold (Table 2), we found that compared to the 2-week threshold, the mean sensitivity for detecting 1-week outbreaks slightly increases in both Tainan and Kaohsiung. In contrast, the mean specificity for detecting 1-week outbreaks decreases, in comparison to the 2-week threshold (Table 3). In addition, the mean values of sensitivity and specificity slightly decrease and increase respectively given *p* value <0.001, compared to *p* value <0.05 (Tables 2, 3). However, the patterns among different parameter combinations are similar, given the different level of *p* value.

## Discussion

Analyzing the routine DF surveillance data from Tainan and Kaohsiung throughout 2014–2015, we leveraged scan statistics and web GIS techniques to design an online analytical tool for local public health workers to promptly monitor the progress of DF and facilitate weekly detection of ongoing transmission at the village level as it had been performed in 2014–2015. Overall, the sensitivity for detecting outbreaks fluctuates dramatically in the beginning and at the end of a DF epidemic, while the specificity of detection behaved inversely (Figs. 6, 7).

Spatial and temporal windows are two major parameters when specifying space–time scan statistics for DF cluster detection [7, 13–15]. The calibration of parameters depends on the complicated interaction between vectors and human beings and therefore requires a process of trial and error. Concerning the temporal window, we found that setting the maximum window from 2 weeks to one month (14–28 days) might be a proper choice based on the existing knowledge of the extrinsic and intrinsic incubation periods of DF [19]. On the other hand, in the absence of DF transmission dynamics information [20] in the study areas, 50% spatial window would be a reasonable maximum.

Our findings further reveal that an adaptive parameter specification would improve the detection accuracy of space–time scan statistics as the disease advanced. A relative large spatial and temporal windows in which more cases were included in the early stage of disease was reasonable since the outbreak of DF was local and sporadic at that moment [21]. In contrast, we would like to shrink both spatial and temporal parameters when the epidemic moves to peak so as to avoid too many false warnings (Figs. 6, 7). Compared to the reference, our covariate adjustment model marginally improves the detection accuracy of sensitivity in Kaohsiung. But the adjusted model (working-age proportion) didn’t work as we expect in Tainan. Whether or not this phenomena is attributed to the different effect on commuting flow patterns between Tainan and Kaohsiung is warranted further study.

Although SaTScan provides geo-referenced files for visualizing hot spots in a GIS platform like ArcGIS (ESRI, Redlands, CA, USA), we integrated the web GIS function into our platform to facilitate visualization in one platform (Fig. 5). In addition, users might explore the possible range of scan statistics parameters interactively and intuitively via the Internet.

This is the first study to apply spatiotemporal scan statistics to prospectively detect DF outbreaks in two major cities of Taiwan. Increased globalization and global warming have been reported to increase DF transmission [22]. The densely populated Southeast Asia region is particularly vulnerable to vector-borne diseases such as DF [23] and the recent Zika virus [24]. We have demonstrated the possibility of detecting vector-borne diseases at the village level on a weekly basis with the minimum data requirement. The method we developed and the web-based tool we created might help local public health workers quickly respond to outbreaks and implement insecticide spraying in the targeted neighborhoods on time in countries or areas prone to DF and Zika. The findings might be a reference for other vector-borne diseases like malaria.

This study has serval limitations. First, our model didn’t consider the true commuting flow patterns due to data unavailability at the village level [25]. Second, as mentioned before, the scan statistics parameters are context dependent, and the estimated range of parameters in the study might not be applicable to cities outside Tainan and Kaohsiung, Taiwan. Third, this study was conducted on retrospectively collected data, and one might encounter problems like data lags or poor data quality in a real-time surveillance situation.

## Conclusions

Using the DF epidemic data of Tainan and Kaohsiung during 2014–2015 as an example, we designed an online scan statistics tool to prospectively detect active DF hot spots. The online scan statistics tool embedding a user-friendly interface and a covariate adjustment model might assist public health works in countries with similar settings in identifying ongoing vector-borne epidemics for targeted interventions.

## Abbreviations

BSA: basic statistical areas
DF: dengue fever
HTML: hypertext markup language
PHP: hypertext preprocessor
TCDC: Taiwan Centers for Disease Control
